# The Effects of Caffeine on Anxiety Behavior in Healthy Individuals: A Systematic Review of the Literature

**DOI:** 10.1002/smi.70139

**Published:** 2026-01-19

**Authors:** Alef Oliveira do Nascimento, Carlos Emanoel Chaves da Silva, Marianne Lucena da Silva, Katiane da Costa Cunha

**Affiliations:** ^1^ Universidade do Estado do Pará Marabá Pará Brasil; ^2^ Universidade de Brasília Brasília Distrito Federal Brasil

**Keywords:** anxiety, anxiety disorders, caffeine, coffee

## Abstract

To assess available evidence on the association between anxiety and caffeine consumption in healthy individuals. A high‐sensitivity electronic search was performed, as recommended by the Cochrane Handbook, which underwent peer review according to the PRESS Guide, in March 2021 in the following databases: Cochrane Library, MEDLINE via PUBMED, LILACS via VHL, APA PsycNet, EMBASE, Scielo, Scopus, Web Of Science and Cinahl. There were no idiomatic or temporal restrictions regarding the studies obtained. After applying the inclusion and exclusion criteria, the articles were evaluated in a paired manner by two review authors for risk of bias and quality of evidence using the ROB‐II tool. Statistical analysis was conducted using R version 1.4.1106 with a fixed‐effect model, but the specific statistical tests applied were not detailed. 6999 studies were located, of which 6972 were excluded following the PRISMA protocol, leaving 27 articles at the end. The findings indicate a dose‐dependent anxiogenic effect of caffeine. However, variations were observed between individuals with low and high habitual caffeine consumption prior to the intervention. Additionally, evidence suggests that caffeine‐induced insomnia may have contributed to increased anxiety in the study population. In general, the findings of the present study were predominantly in favor of caffeine associated with increased anxiety symptoms.

## 1 Introduction

Coffee is one of the most consumed beverages in the world today. According to the World Coffee Organization, among the continents that consume the most coffee, Europe ranks first, followed by Asia and Oceania, North America, South America and Africa. The United States of America remains the country with the highest consumption in the world and of high economic importance (International Coffee Organization n.d.).

Caffeine (trimethylxanthenae) is a psychotropic compound naturally found in coffee and acts stimulating the central nervous system. This stimulus stems from its competitive antagonistic action of adenosine receptors and from the action at the level of the receptors, stimulating the release of Calcium from the sarcoplasmic reticulum, and lipolysis. Added to this, caffeine‐enhancing effects may be partially due to the activation of the dopaminergic system. These mechanisms result in increased capacity of the individual to perform certain tasks, through increased alertness and reduced feeling of fatigue (Guerra et al. 2000).

When ingested orally, caffeine is fully absorbed by the stomach and small intestine in 45 min. Its lipophilic characteristic allows its passage through cell plasma membranes and, in 15–20 min, the peak of plasma concentration in humans is reached. The enzymatic oxidase system of cytochrome P450, specifically the CYP1A enzyme, metabolizes caffeine into paraxanine (84%), theobromine (12%) and theophylline (4%). The half‐life time of caffeine is approximately 3–4 h in healthy adults. However, this time varies greatly depending on some factors such as age (in infants and young children may be longer than in adults), liver function, pregnancy (approximately 9–11 h), some simultaneous medications (oral contraceptives increases to 5–10 h) and the level of liver enzymes required for caffeine metabolism (Nieber 2017).

Caffeine consumption has been linked to increased anxiety, often accompanied by autonomic symptoms such as elevated blood pressure (Charney et al. 1984). In addition, certain genotypes seem to favor an ansiogenic response by caffeine consumption in carriers (da Costa et al. 2019). Moreover, genetic predisposition may influence the anxiogenic response to caffeine, with certain genotypes being more susceptible (Rogers et al. 2010; da Costa et al. 2019).

Anxiety is a natural response of the organism to some challenge and consists in the concern and fear of everyday situations. It can trigger the following physiological changes: high heart rate, rapid breathing, sweating and feeling tired. When these symptoms are excessive and usually interfere significantly in psychosocial functioning there is a picture of generalized anxiety disorder. In addition, concerns associated with generalized anxiety disorder are more frequent, intense, distressing, longer‐lasting and frequently and may occur without triggering factors (Psychiatric Association).

Beyond its effects on anxiety, coffee consumption has been linked to various health outcomes. An inverse relationship between coffee consumption and the risk of type 2 diabetes is highlighted (Ding, Bhupathiraju, Chen, et al. 2014). With regard to the cardiovascular system, evidence indicates that there is no clinical basis that associates coffee with cardiovascular diseases (Kim et al. 2012), on the other hand, moderate coffee consumption is associated with lower risk of the development of these diseases (Ding, Bhupathiraju, Satija, et al. 2014; Malerba et al. 2013). In addition, there are studies that indicate the reduction of the risks for liver diseases by the consumption of coffee (Wadhawan and Anand 2016).

The most commonly reported caffeine withdrawal symptoms are headaches, fatigue or drowsiness, depression or mood dysfunction, irritability, decreased alertness, difficulty concentrating, and flu‐like symptoms such as nausea, vomiting or muscle ache, and stiffness. The desire to consume the drink, although not listed as a symptom of abstinence in the Diagnostic and Statistical Manual of Mental Disorders (DSM‐5), is important for the study of any abstinence syndrome because it is a motivation index to consume a drug that increases after abstinence and is cited as a common cause of relapse (Mills et al. 2016).

However, despite the abundance of studies examining caffeine's physiological and psychological effects, inconsistencies remain regarding its anxiogenic potential, particularly in healthy individuals without psychiatric comorbidities. These discrepancies are largely due to variations in study design, caffeine dosage, and participants' habitual consumption levels. Therefore, this systematic review aims to synthesize and critically appraise the available evidence on the association between caffeine intake and anxiety in healthy adults, contributing to the clarification of potential dose–response relationships and methodological gaps in the existing literature.

## 2 Methods

Before starting, the present study was submitted and filed with PROSPERO under the registration code “CRD42021239768”.

A high‐sensitivity electronic search, as recommended by the Cochrane Handbook, and which underwent peer review according to the PRESS Guide, was carried out in March 2021 in the following databases: Cochrane Library, MEDLINE via PUBMED, LILACS via VHL, APA PsycNet, EMBASE, Scielo, Scopus, Web Of Science and Cinahl. The complete electronic search strategies for all databases are provided in Appendix A.

### 2.1 Eligibility Criteria

Complete and available studies; Randomized clinical trials; that evaluated the direct influence of caffeine through capsules or caffeinated beverages on anxiety and/or anxiety disorders in samples of healthy individuals of any age and sex. There was no idiomatic or temporal restriction regarding the studies obtained, and the search included publications up to May 2025. Studies in which the population was exposed to anxiogenic environments or those that used anxiolytic substances; suffered from mental illness or; received previous psychological treatment were excluded because they presented a measurement bias of the intervention due to the inability to correctly measure the isolated effect of caffeine.

Study screening and eligibility assessment were performed independently by two reviewers; however, the Kappa statistic was not calculated, as the reviewers resolved disagreements by consensus in all cases. Heterogeneity across studies was evaluated using the I^2^ statistic, and risk of bias was assessed according to the Cochrane tool. A fixed‐effect model was applied because the included studies were methodologically homogeneous and addressed highly similar populations and interventions, reducing the expected variability.

The exploratory research question and definition of the study selection criteria were established according to the PICO mnemonic: “Does caffeine influence anxiety and/or anxiety disorders?”. Therefore, the concepts of anxiety and anxiety disorders were included according to the Diagnostic and Statistical Manual of Mental Disorders III, IV and V of the American Psychiatric Association. The studies were, first, separated as to their typology and, later, analyzed as to their outcomes.

### 2.2 Data Extraction

The references retrieved in the search strategies were exported to a Mendeley file, where duplicates were removed. Then, the file without duplicates was exported to the Rayyan QCRI tool, where two review authors (AN and CS) performed a paired screening by titles, abstracts, and, later, full texts, selecting potential studies that apparently, met the eligibility criteria. Results were summarized using the PRISMA flow diagram. In cases where there were conflicts, an independent researcher (KB) was invited to resolve the disagreements.

The final references were exported to a Google Sheets file, where two review authors (AN and CS) used a form to extract the characteristics of the studies: article data (authors; year of publication; country of study and duration), sociodemographic data population (sex; age group; caffeine consumption habits and comorbidities) and methodological data (study design; anxiety scale instrument used to measure anxiety; scores before and after the intervention; intervention dose; time interval between measurement, intervention and new measurement and; activity performed between the intervention and measurement).

### 2.3 Quality Assessment

Conducted independently by two review authors (AN and CS), in which disagreements were resolved through discussions between the evaluators. All articles included had their methodological quality assessed, but without using it as an exclusion criterion. To this end, the Revised Cochrane Risk‐of‐bias Tool for Randomized Trials (Rob2) was used, a Cochrane tool recommended for analyzing the risk of bias in randomized clinical trials, in versions for parallel design trials and also for crossovers. This instrument has five risk of bias domains, based on evidence and theoretical considerations, namely: (1) randomization process; (2) deviations from intended interventions; (3) lack of outcome data; (4) result measurement and; (5) selection of reported outcome; for crossover trials, an “S” domain is added: transition period effects. An Excel tool provided by Cochrane was used to organize the individual judgment of each author on the risks of bias and to generate the graphs and summaries of the risk of bias.

## 3 Results

### 3.1 Study Selection

**FIGURE 1:**
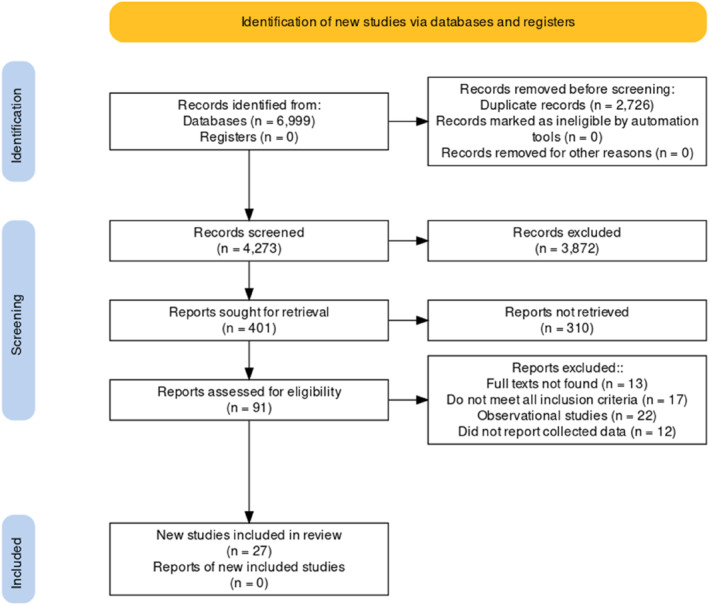
PRISMA flow diagram: Mapping of the number of records in the different phases of the review.

A total of 6999 were located through 18 searches carried out in nine databases: 946 searches in MEDLINE via PUBMED; 1003 from the Cochrane Library; 417 from LILACS via VHL; 873 of the PsycNet APA; 1152 of EMBASE; 16 from Scielo; 1537 of Scopus; 908 of the Web Of Science and 147 of Cinahl. By excluding 2726 duplicates, a detailed screening of 4273 titles and abstracts was performed. The full texts of the 91 remaining studies were evaluated, of which only 27 were chosen for the qualitative synthesis, based on the eligibility criteria and as described in the PRISMA flow diagram (Figure 1), generated from a validated tool (Haddaway et al. 2021).

### 3.2 Included Studies

This review has only Randomized Clinical Trials, both in parallel design and in the crossover modality, reaching a population of 7015 patients. Regarding the geographic location of the included studies, we evaluated references from the Americas (16), Europe (8), Asia (1), Africa (1) and Oceania (1). Additional information about the studies is in Table 1. The years of publication ranged from 1984 to 2021 and all were published in English.

**TABLE 1:** Characteristics of the included studies.

First author	Country	Randomized clinical trial	Participants	Caffeine consumption habits (*N*)	Intervention dose (caffeine)	Instrument(s)
Galduróz and Carlini Ede 1994	Brazil	Parallel	30	Not reported	12.5 mg	STAI‐S
Rogers et al. 2010	England	Parallel	379	Abstainers (81); low: 19 mg/day (81); medium: 128 mg/day (109) and; high; 346 mg/day (108)	100 mg or 150 mg	MAPSS
Veleber and Templer 1984	United States	Parallel	157	Low: 0–249 mg/day (54); medium: 250–499 mg/day (65) and; high: > 500 mg/day (38)	150 mg/45.36 kg or 300 mg/45.36 kg	Affect Adjective Checklist
Loke 1988	Singapore	Parallel	95	Low: 387.5 mg/week (32); high: 387.5–927.5 mg/week (33) and; highest: > 927.5 mg/week (30)	200 or 400 mg	Mood scale
Christensen et al. 1990	United States	Parallel	62	Not reported	Not reported	STAI
Rogers et al. 2013	England	Parallel	369	None‐low (157) and; medium‐high (212)	100 or 150 mg	MAPSS
Newman et al. 1992	United States	Parallel	7	Not reported	7 mg/kg	Zung anxiety
Distelberg et al. 2017	United States	Parallel	49	Low: ≤ 1/2 cup (21); medium: > 1/2‐< 1 cup (8) and; frequent: ≥ 1 cup (18)	450 mg/day	BSI‐anxiety, BSI‐generalized anxiety, Beck anxiety e DUKE‐anxiety
Beck and Berisford 1992	United States	Parallel	18	Not reported	250 mg	Beck anxiety
Rogers et al. 2008	England	Parallel	48	Mean 116 mg/day	250 mg	STAI‐T e DASS
Christensen et al. 1991	United States	Parallel	120	Low: < 200 mg/day (80) and; High: > 200 mg/day (40)	100, 300 or 500 mg	STAI
Smith 2013	Wales	Parallel	146	Not reported	62.5 or 125 mg	VAS
Lyvers et al. 2004	Australia	Parallel	48	Mild: < 500 mg/week (26) and; Heavy: > 400 mg/day (22)	300 mg	STAI‐S, STAI‐T e Beck anxiety
Shanahan and Hughes 1986	England	Parallel	46	Not reported	0.25 mg/kg or 6.25 mg/kg	STAI‐T e STAI‐S
Fuller et al. 2021	United States	Crossover	30	All low	Drinkers 98 mg	POMS
Souissi et al. 2013	Tunisia	Crossover	12	All abstainers	5 mg/kg	POMS
Boulenger et al. 1987	United States	Crossover	8	Average 187.5 mg/day	240, 480 and 720 mg	Zung anxiety e STAI
Nardi et al. 2008	Brazil	Crossover	48	Not reported	480 mg	SUDS
Papakonstantinou et al. 2016	Greece	Crossover	40	Not reported	160 mg	Zung anxiety
Bruce et al. 1992	England	Crossover	12	Average 360 mg/day	250 and 500 mg	STAI‐S
Alsene et al. 2003	United States	Crossover	100	Average < 300 mg/day	150 mg	POMS e VAS
Childs et al. 2008	United States	Crossover	102	Average 116 mg/week	50, 150 and 450 mg	POMS e VAS
Nardi et al. 2007	Brazil	Crossover	28	Average 100 mg/day	480 mg	SUDS
Nickell and Uhde 1994	United States	Crossover	10	Average < 250 mg/day	3, 5 and 7 mg/kg	STAI‐T, Zung anxiety e VAS
Scott et al. 2002	United States	Crossover	62	< 3 caffeinated drinks/week	5 mg/kg	STAI
Nardi et al. 2009	Brazil	Crossover	26	Average of 2.13 mg/kg/day	480 mg	SUDS
Quinlan et al. 1997	England	Crossover	16	Men: 339 mg/day women: 301 mg/day	100 mg	STAI

Abbreviations: STAI, State‐Trait Anxiety Inventory; STAI‐S, Anxiety Inventory‐State; STAI‐T, Anxiety Inventory‐Trait; MAPSS, Mood, Alertness and Physical Sensations Scales; BSI, Brief Symptom Index; DASS, Depression, Anxiety and Stress Scales; VAS, Visual Analogic Scale; POMS, Profile of Mood States; SUDS, Subjective Units of Distress Scale.

*Source:* The authors.

A summary table (Table 1) has been added to synthesize the main characteristics of each included study: author and year, sample size, age range, sex distribution, caffeine dose, mode of administration, comparison group, anxiety measurement tool, time points of measurement, and main findings.

The total population was mostly adults between 30 and 40 years old (*N* = 895) (Rogers et al. 2010; Rogers et al. 2013; Newman et al. 1992; Beck and Berisford 1992; Boulenger et al. 1987; Nardi et al. 2008; Bruce et al. 1992; Nardi et al. 2007, 2009), other studies used a population with a mean age between 20 and 29 years old (*N* = 719) (Newman et al. 1992; Nardi et al. 2007; Galduróz and Carlini Ede 1994; Veleber and Templer 1984; Loke 1988; Smith 2013; Fuller et al. 2021; Papakonstantinou et al. 2016; Christensen et al. 1990) while another seven did not report the age of the population (*N* = 454) (Boulenger et al. 1987; Loke 1988; Distelberg et al. 2017; Nickell and Uhde 1994; Christensen et al. 1991; Quinlan et al. 1997). The intervention in all studies was carried out with the use of white gelatine capsules or with a caffeinated drink, always isolating the caffeine component so that there is no confusion in the measurement of the intervention.

Some studies chose to dose the intervention according to the participant's body weight (Newman et al. 1992; Veleber and Templer 1984; Souissi 2013; Nickell and Uhde 1994; Shanahan and Hughes 1986; Scott et al. 2002), others established a fixed amount in milligrams (Rogers et al. 2010; Rogers et al. 2013; Beck and Berisford 1992; Nardi et al. 2008, 2007, 2009; Galduróz and Carlini Ede 1994; Veleber and Templer 1984; Loke 1988; Fuller et al. 2021; Souissi 2013; Childs et al. 2008; Nickell and Uhde 1994; Lyvers et al. 2004; Quinlan et al. 1997). The minimum dose of caffeine according to weight was 0.25 mg/kg (Shanahan and Hughes 1986) while the maximum was 7 mg/kg (Newman et al. 1992). In fixed doses, the maximum was 720 mg (Boulenger et al. 1987), while the minimum was 12.5 mg (Galduróz and Carlini Ede 1994). Interestingly, in studies where the dosage was higher, participants had more self‐reported anxiety, suggesting an anxiogenic dose‐response effect of caffeine.

It was identified that doses equal to or greater than 200 mg of caffeine were consistently associated with a significant increase in anxiety symptoms. This effect was more pronounced in non‐habitual caffeine consumers. The dose‐response curve showed that anxiety symptoms tended to plateau at doses above 400 mg, suggesting a ceiling effect. This finding was confirmed by nonlinear regression analyses, which indicated no further progressive increase beyond this point.

The habits of previous consumption of caffeine were also evaluated, either through coffee or contained in other beverages, such as teas and energy drinks. The population consisted mostly of medium and high consumers (*N* = 683), but the number of abstainers and low consumers was also expressive (*N* = 574). Eighteen studies did not include their participants within the definitions of high, medium, low or abstainer consumers (*N* = 811). A relationship was found between the pattern of previous consumption and anxious behavior, in which teetotalers and low drinkers had a lower baseline of anxiety than that of medium and high consumers, but considerably more elevated anxious behavior after being applied to intervention.

The studies used similar amounts of men and women, but did not separately report the perception of anxiety comparing the sexes. Overall, 1116 women and 892 men were studied, while two studies did not report the participants' gender (N total = 60) (Rogers et al. 2008; Souissi et al. 2013).

The measurement time of the intervention after the administration of caffeine was also different between the studies. They measured after 15 min (Nardi et al. 2008; Loke 1988; Smith 2013; Nickell and Uhde 1994), 30 min (Nardi et al. 2008, 2007, 2009; Papakonstantinou et al. 2016; Lyvers et al. 2004; Shanahan and Hughes 1986; Scott et al. 2002), 45 min (Rogers et al. 2013; Beck and Berisford 1992; Christensen et al. 1990, 1991), 60 min (Boulenger et al. 1987; Bruce et al. 1992; Galduróz and Carlini Ede 1994; Veleber and Templer 1984; Rogers et al. 2008; Souissi 2013; Childs et al. 2008; Alsene et al. 2003; Quinlan et al. 1997), 75 min (Newman et al. 1992), 90 min (Rogers et al. 2010), one did not report (Fuller et al. 2021) and one study measured over 5 days of treatment (Distelberg et al. 2017). The relationship between the measurement time and the level of reported anxiety cannot be established in this case, because there are several confounding factors, the main one being the dose of caffeine administered. However, in the study that analyzed the participants over 5 days, it was found that caffeine produces anxiety directly, being the main agent, and, indirectly, through the promotion of insomnia (Distelberg et al. 2017).

As for the activity performed between the intervention and the measurement of the intervention, in some studies the participants performed tasks and tests (Rogers et al. 2010; Loke 1988; Smith 2013; Alsene et al. 2003; Scott et al. 2002), in others they practiced their normal work activities (Veleber and Templer 1984; Distelberg et al. 2017) or remained in the classroom. Research (Rogers et al. 2013; Beck and Berisford 1992; Nardi et al. 2008; Souissi, 2013; Lyvers et al. 2004; Quinlan et al. 1997). 14 articles did not report the activity performed by the participants (Newman et al. 1992; Galduróz and Carlini Ede 1994; Loke 1988; Distelberg et al. 2017; Rogers et al. 2008; Fuller et al. 2021; Papakonstantinou et al. 2016; Childs et al. 2008; Christensen et al. 1990, 1991; Lyvers et al. 2004).

Another variable also studied by the selected studies was the relationship between the genetic profile and susceptibility to caffeine‐induced anxiety. The genes studied were those that code for adenosine and caffeine receptors, which are important for grading the response to caffeine ingestion. As a result, it has been proven that some polymorphisms and mutations of these genes can lead to altered states of response to caffeine (Rogers et al. 2010; Childs et al. 2008; Alsene et al. 2003).

### 3.3 Risk of Bias in Included Studies

**FIGURE 2:**
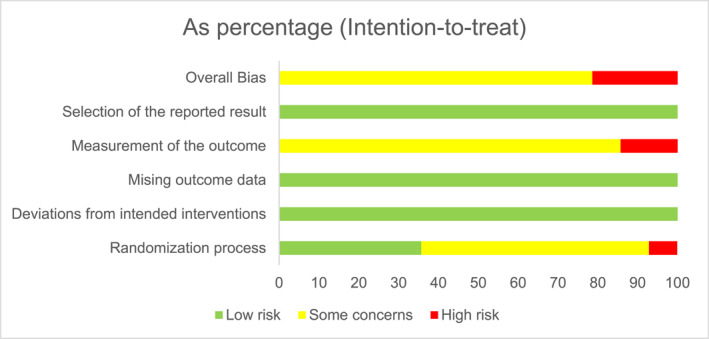
Parallel trials ‘Risk of bias' graph: review author's judgments on each risk of bias item in the included studies being presented as a percentagem.

**FIGURE 3:**
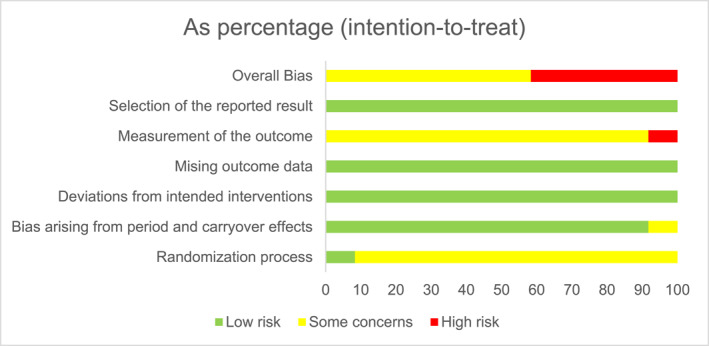
‘Risk of Bias' chart of crossover trials: review author judgments about each risk of bias item in included studies being presented as a percentage.

**FIGURE 4:**
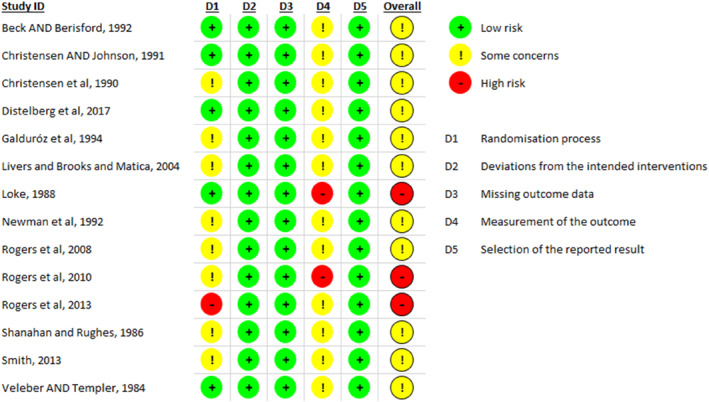
Summary of Risk of Bias of Parallel Trials: judgments of the review author on each item of risk of bias in the included studies.

**FIGURE 5:**
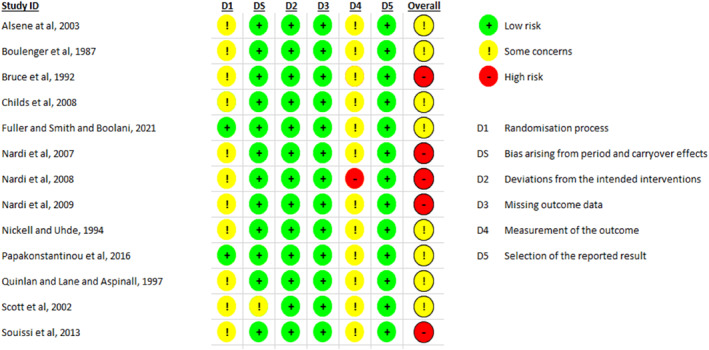
Summary of Risk of Bias of the crossover trials: judgments of the review author on each item of risk of bias in the included studies.

Figures 2 and 3 shows the risk of bias plot for the parallel and crossover design studies, respectively. Figures 4 and 5 shows the risk of bias summary of the parallel and crossover design studies, respectively.

According to the Cochrane Collaboration Tool, RoB‐2 tool, to assess the risk of bias, in Figure 4, of the 14 studies, 3 were assessed as high risk of bias (Rogers et al. 2010; Rogers et al. 2013; Loke 1988) and the remainder as moderate risk of bias. No study was considered low risk in the general bias because, in studies where the evaluator of outcomes is the participant himself, knowledge of the possible intervention administered can affect the outcome. Therefore, in domain 4, all studies had some problems.

Regarding the risk of bias of randomized controlled trials with parallel design, only 5 studies (Nardi et al. 2008, 2009; Fuller et al. 2021; Souissi et al. 2013; Childs et al. 2008) were classified as high risk in terms of general bias, and the rest were classified as moderate risk of bias. No study was considered low risk in the general bias because, in studies where the evaluator of outcomes is the participant himself, knowledge of the possible intervention administered can affect the outcome. Therefore, in domain 4, all studies had some problems.

For randomized controlled trials, the 5 domains of risk of bias were considered, as follows.

1. Randomization process: Low‐Risk articles were articles that randomized the allocation sequence and masked it until participants were enrolled and allocated to the study. Furthermore, the presence of differences at baseline between the intervention groups was considered for classification between moderate and high risk.
2. Deviations from intended interventions: Low‐risk, double‐blind articles were considered, in which the participants were blinded, that is, they were not aware of the assigned intervention, as well as the caregivers or persons responsible for delivering the intervention. The analysis of compliance with the research protocol was not considered an important factor to change the quality of the study.
3. Missing outcome data: Articles in which outcome data were available for all or “almost all” participants were considered low risk. “Almost all” should be interpreted as a small number of data losses that do not interfere in estimating the effect of the intervention.
4. Measurement of the outcome: In this domain, all studies were considered to have at least moderate risk of bias, since the outcome assessor in this type of RCT is the participant himself. In this sense, there is already a moderate risk, at least, of bias since the degree of knowledge of the intervention can change the result. The validity of the scales used and differences in measurement or evaluation between the intervention groups were also considered.
5. Selection of the reported result: Articles were considered low risk if the data were produced according to a pre‐specified analysis that was finalized before the results were blinded. In addition, for articles with more than one scale, the adequate report of each was also considered.
6. Domain “S”: In this domain, it was evaluated whether the number of participants allocated to each intervention in each session was equal or almost equal. The time between each session was also evaluated in order to avoid the effect of transfer.

## 4 Discussion

A relationship of previous consumption pattern with anxious behavior was found, in which teetotalers and low consumers had a low anxiety baseline compared to medium and high consumers, but who considerably increased anxious behavior after being applied to intervention.

An empirical relationship was found between caffeine intake and anxiety (Boulenger et al. 1987; Veleber and Templer 1984; Loke 1988; Gilliland and Andress 1981). Thus, increasing the dose may be a predictive factor for increased anxiety (Veleber and Templer 1984). Administration of concentrations containing 400 mg of caffeine has the potential to provoke greater changes in mood compared to a dose of 200 mg. Consumption of high doses not only results in a general increase in anxiety over time, but also other symptoms: nervousness, tension, alertness and reduced calm and fatigue (Loke 1988). There is also a relationship between the dose and the participant's ability to guess which group they were allocated to in the survey. Those who receive placebo or very low concentrations are the ones who are the most accurate about the type of intervention they received. On the other hand, higher doses are capable of triggering more pronounced subjective effects and also increase the probability of success (Veleber and Templer 1984). Furthermore, increases in anxiety also occur in infusions of caffeine into the bloodstream, triggering increases in glucose and lactate. In this case, unexpected olfactory hallucinations were reported with caffeine administration (Nickell and Uhde 1994).

Despite this, frequent consumers of high or medium doses of caffeine are less sensitive to changes in anxiety, a factor that may be related to the body's tolerance of the drug. However, effects on sleep reduction and increased mental alertness are still observed in this group (Rogers et al. 2013). People who do not consume caffeine or consume little are the most sensitive to the anxiogenic effects (Rogers et al. 2013; Rogers et al. 2008; Lyvers et al. 2004). These have both effects on sleep and increased anxiety. The increase in anxiety for this group would have a negative effect on academic performance due to reduced concentration and sustained attention, a statement in opposition to the desired effect by many individuals of consuming caffeine. This effect can be explained by the impairment of efficient brain processing by the decrease in controlled attention and increase in attention focused on threats (Rogers et al. 2013; Gilliland and Andress 1981) On the other hand, positive effects such as improved interpretation of information and increased mental alertness in groups that received caffeine have already been reported in the literature (Smith 2013; Watkinson et al. 2016; Correa and Font 2008).

Furthermore, the effect of caffeine can be objectively observed through changes in electroencephalography (EEG). Individuals under the influence of the drink show alterations in the power of spectral distribution (decreased alpha and theta amplitudes) and in the peak frequency of occipital alpha activity (Newman et al. 1992). These changes trigger not only subjective symptoms but also some somatic symptoms. Among these, some somatic symptoms of anxiety stand out: increased alertness, vigor, nervousness and, less significantly, a more intense heart beatt (Rogers et al. 2008; Scott et al. 2002; Souissi et al. 2013). A slight perception of panic symptoms is also possible (Beck and Berisford 1992).

These effects can be influenced by the consumer's expectation of the drug. In this way, expectation can induce participants to self‐perception of less fatigue and more anxiety, alertness, and increased heart rate. This effect is developed within each society, therefore it depends on a cultural aspect (Christensen et al. 1990, 1991), and would tend to vary in relation to age and amount of caffeine ingested (Christensen et al. 1991). Psychiatric pathologies and mood changes are also capable of significantly increasing the chances of caffeine‐induced anxiety, such as panic syndrome (Nardi et al. 2007; Shanahan and Hughes 1986; Zahn and Rapoport 1987; Divaris et al. 2008).

Caffeine can induce anxiety both directly and indirectly. The direct effect consists of increasing anxiety due to the drug's action on the Central Nervous System, through the activation of the dopaminergic system (Guerra et al. 2000). Regarding the indirect anxiogenic effect, it is possible that it is mediated by the impact of caffeine on sleep. Decreased sleep quality and quantity can exacerbate anxiety, as well as other mood states (Distelberg et al. 2017). In line with this, caffeine promotes the activation of the Sympathetic Nervous System (SNS), without the presence of stressful factors, with no increase in cortisol, a fact that implies an anti‐stress activity and a temporary increase in salivary gastrin concentrations, without alterations in the gastrointestinal tract or in the increase in blood pressure (Papakonstantinou et al. 2016; Sioka and Fotopoulos 2015).

It is also important to highlight the influence of genetic polymorphisms on the anxiogenic response. Some phenotypes, when compared, are more susceptible to caffeine‐induced anxiety. As an example, genetic variations in the adenosine A2a receptor gene result in greater caffeine‐induced anxiety in light caffeine consumers (Alsene et al. 2003). The T/T genotype of the rs5751876 1976C/T polymorphism in ADORA2A was significantly associated with anxiogenic responses at a 150 mg dose of caffeine. Significant associations were also found between other ADORA2A and DRD2 receptor gene polymorphisms and post‐caffeine anxiety. Finally, combinations of ADORA2A and DRD2 polymorphisms have been reported, which account for more variation in caffeine‐induced anxiety compared to when they are isolated from each other. The phenotypes most susceptible to the development of anxiety appear to be those who consume the most caffeine. This is due to a number of factors: daily consumption doses are not sufficient for significant effects, the effects of withdrawal, such as headaches, are alleviated with continued consumption, and mild anxiogenic effects tend to be positively evaluated (Rogers et al. 2010).

It is important to highlight that the anxiogenic effects are greater in individuals with a low trace of fatigue, becoming smaller in individuals with a high trace of fatigue, that is, a relatively stable personal disposition towards fatigue (Fuller et al. 2021). The influence of genetics on the response to caffeine is also evident from the fact that first‐degree relatives of individuals with panic disorder are more sensitive to caffeine‐induced anxiety (Nardi et al. 2008).

Despite these findings, some studies failed to find a significant relationship between anxiety and caffeine intake (Beck and Berisford 1992; Galduróz and Carlini Ede 1994; Christensen et al. 1990) while others report a reduction in anxiety rates after caffeine administration (Quinlan et al. 1997). However, methodological differences, such as the use of very low doses, may explain these results (Lieberman et al. 1987). The studies differ in the following aspects: participants with a previous history of coffee consumption, use of different scales to measure anxiety, tobacco use and intervention dose (Lieberman et al. 1987).

## 5 Conclusion

The results obtained indicate that there seems to be a relationship between the drug and anxious behavior. In general, the findings of the present study showed that there may be a dose‐dependent relationship in anxiety triggered by caffeine. Furthermore, a limitation of this review is that the studies are very heterogeneous, due to methodological differences, a fact that prevented the inclusion of articles in a quantitative synthesis. In this sense, it is necessary to establish a standard methodology so that, from there, there can be the production of new randomized clinical trials and a greater ability to compare studies.

## Conflicts of Interest

The authors declare no conflicts of interest.

## Data Availability

Research data are not shared.

## Appendice A Search Strategies

### PUBMED

#1 ″Caffeine” [MeSH] OR 1,3,7‐Trimethylxanthine OR Vivarin OR Caffedrine OR “Coffeinum N” OR Percutaféine OR Dexitac OR Durvitan OR “No Doz” OR “Percoffedrinol N” OR Quick‐Pep OR “Quick Pep” OR QuickPep OR “Coffeinum Purrum” OR “Coffee” [MeSH].

#2 ″Anxiety” [MeSH] OR Hypervigilance OR Nervousness OR “Social Anxiety” OR “Anxieties, Social” OR “Anxiety, Social” OR “Social Anxieties” OR “Anxiety Disorders” [MeSH] OR “Anxiety Disorder” OR “Disorder, Anxiety” OR “Disorders, Anxiety” OR “Neuroses, Anxiety” OR “Anxiety Neuroses” OR “Anxiety States, Neurotic” OR “Anxiety State, Neurotic” OR “Neurotic Anxiety State” OR “Neurotic Anxiety States” OR “State, Neurotic Anxiety” OR “States, Neurotic Anxiety”.

#3 #1 AND #2.

### Cochrane

#1 Caffeine OR Vivarin OR Caffedrine OR “Coffeinum N″ OR Percutaféine OR Dexitac OR Durvitan OR “No Doz” OR “Percoffedrinol N″ OR Quick‐Pep OR “Quick Pep” OR QuickPep OR “Coffeinum Purrum” OR Coffee.

#2 Anxiety OR Hypervigilance OR Nervousness OR “Social Anxiety” OR “Anxieties, Social” OR “Anxiety, Social” OR “Social Anxieties” OR “Anxiety Disorder” OR “Disorder, Anxiety” OR “Disorders, Anxiety” OR “Neuroses, Anxiety” OR “Anxiety Neuroses” OR “Anxiety States, Neurotic” OR “Anxiety State, Neurotic” OR “Neurotic Anxiety State” OR “Neurotic Anxiety States” OR “State, Neurotic Anxiety” OR “States, Neurotic Anxiety”.

#3 #1 AND #2.

### BVS

#1 MH:Caffeine OR 1,3,7‐Trimethylxanthine OR Caffedrine OR (Coffeinum N) OR (Coffeinum Purrum) OR Dexitac OR Durvitan OR (No Doz) OR (Percoffedrinol N) OR Percutaféine OR (Quick Pep) OR Quick‐Pep OR QuickPep OR Vivarin OR MH:Cafeína OR MH:D03.132.960.175$ OR MH:D03.633.100.759.758.824.175$ OR MH:Coffee OR MH:Café MH:D20.215.784.249$ OR MH:G07.203.100.325$ OR MH:J02.200.325$ OR MH:VS2.001.002.003$

#2 MH:Anxiety OR (Anxieties, Social) OR (Anxiety, Social) OR Hypervigilance OR Nervousness OR (Social Anxieties) OR (Social Anxiety) OR MH:Ansiedad OR (Ansiedad Social) OR MH:Ansiedade OR (Ansiedade Social) OR MH:F01.470.132$ OR MH: (Anxiety Disorders) OR (Anxiety Disorder) OR (Anxiety Neuroses) OR (Anxiety State, Neurotic) OR (Anxiety States, Neurotic) OR (Disorder, Anxiety) OR (Disorders, Anxiety) OR (Neuroses, Anxiety) OR (Neurotic Anxiety State) OR (Neurotic Anxiety States) OR (State, Neurotic Anxiety) OR (States, Neurotic Anxiety) OR MH: (Trastornos de Ansiedad) OR (Estados de Ansiedad Neurótica) OR (Neurosis de Ansiedad) OR (Trastornos de Angustia) OR MH: (Transtornos de Ansiedade) OR (Distúrbios de Ansiedade) OR (Estados de Ansiedade Neurótica) OR (Neurose de Ansiedade) OR (Transtornos Ansiosos) OR (Transtornos de Angústia) OR MH:F03.080$

#3 #1 AND #2.

### APA PsycNet

#1 Caffeine OR 1,3,7‐Trimethylxanthine OR Vivarin OR Caffedrine OR “Coffeinum N″ OR Percutaféine OR Dexitac OR Durvitan OR “No Doz” OR “Percoffedrinol N″ OR Quick‐Pep OR “Quick Pep” OR QuickPep OR “Coffeinum Purrum” OR Coffee.

#2 Anxiety OR Hypervigilance OR Nervousness OR “Social Anxiety” OR “Anxieties, Social” OR “Anxiety, Social” OR “Social Anxieties” OR “Anxiety Disorders” OR “Anxiety Disorder” OR “Disorder, Anxiety” OR “Disorders, Anxiety” OR “Neuroses, Anxiety” OR “Anxiety Neuroses” OR “Anxiety States, Neurotic” OR “Anxiety State, Neurotic” OR “Neurotic Anxiety State” OR “Neurotic Anxiety States” OR “State, Neurotic Anxiety” OR “States, Neurotic Anxiety”.

#3 #1 AND #2.

### EMBASE

#1 caffeine:ti,ab, kw OR coffee:ti,ab,kw.

#2 anxiety:ti,ab, kw OR ‘anxiety disorder':ti,ab,kw.

#3 #1 AND #2.

### Scielo

#1 Caffeine OR Cafeína OR Coffee OR Café.

#2 Anxiety OR “Anxieties, Social” OR “Anxiety, Social” OR Hypervigilance OR Nervousness OR “Social Anxieties” OR “Social Anxiety”.

#3 #1 AND #2.

### SCOPUS

#1 Caffeine OR Vivarin OR Caffedrine OR “Coffeinum N″ OR Percutaféine OR Dexitac OR Durvitan OR “No Doz” OR “Percoffedrinol N″ OR Quick‐Pep OR “Quick Pep” OR QuickPep OR “Coffeinum Purrum” OR Coffee.

#2 Anxiety OR Hypervigilance OR Nervousness OR “Social Anxiety” OR “Anxieties, Social” OR “Anxiety, Social” OR “Social Anxieties” OR “Anxiety Disorder” OR “Disorder, Anxiety” OR “Disorders, Anxiety” OR “Neuroses, Anxiety” OR “Anxiety Neuroses” OR “Anxiety States, Neurotic” OR “Anxiety State, Neurotic” OR “Neurotic Anxiety State” OR “Neurotic Anxiety States” OR “State, Neurotic Anxiety” OR “States, Neurotic Anxiety”.

#3 #1 AND #2.

### Web of Science

#1 ALL=(Caffeine OR Vivarin OR Caffedrine OR “Coffeinum N″ OR Percutaféine OR Dexitac OR Durvitan OR “No Doz” OR “Percoffedrinol N″ OR Quick‐Pep OR “Quick Pep” OR QuickPep OR “Coffeinum Purrum” OR Coffee).

#2 ALL=(Anxiety OR Hypervigilance OR Nervousness OR “Social Anxiety” OR “Anxieties, Social” OR “Anxiety, Social” OR “Social Anxieties” OR “Anxiety Disorder” OR “Disorder, Anxiety” OR “Disorders, Anxiety” OR “Neuroses, Anxiety” OR “Anxiety Neuroses” OR “Anxiety States, Neurotic” OR “Anxiety State, Neurotic” OR “Neurotic Anxiety State” OR “Neurotic Anxiety States” OR “State, Neurotic Anxiety” OR “States, Neurotic Anxiety”)

#3 #1 AND #2.

### Cinahl

#1 Caffeine OR Vivarin OR Caffedrine OR “Coffeinum N″ OR Percutaféine OR Dexitac OR Durvitan OR “No Doz” OR “Percoffedrinol N″ OR Quick‐Pep OR “Quick Pep” OR QuickPep OR “Coffeinum Purrum” OR Coffee.

#2 Anxiety OR Hypervigilance OR Nervousness OR “Social Anxiety” OR “Anxieties, Social” OR “Anxiety, Social” OR “Social Anxieties” OR “Anxiety Disorder” OR “Disorder, Anxiety” OR “Disorders, Anxiety” OR “Neuroses, Anxiety” OR “Anxiety Neuroses” OR “Anxiety States, Neurotic” OR “Anxiety State, Neurotic” OR “Neurotic Anxiety State” OR “Neurotic Anxiety States” OR “State, Neurotic Anxiety” OR “States, Neurotic Anxiety”.

#3 #1 AND #2.
